# Management of Multiple Arteriovenous Malformations of the Small Bowel

**DOI:** 10.1155/2019/2046857

**Published:** 2019-12-11

**Authors:** Masahiro Hirakawa, Rie Ishizuka, Masanori Sato, Naotaka Hayasaka, Hiroyuki Ohnuma, Kazuyuki Murase, Kohichi Takada, Tatsuya Ito, Takayuki Nobuoka, Koji Miyanishi, Masayoshi Kobune, Ichiro Takemasa, Junji Kato

**Affiliations:** ^1^Department of Medical Oncology, Sapporo Medical University School of Medicine, Sapporo, Japan; ^2^Department of Surgery, Surgical Oncology and Science, Sapporo Medical University School of Medicine, Sapporo, Japan; ^3^Department of Hematology, Sapporo Medical University School of Medicine, Sapporo, Japan

## Abstract

A 62-year-old Japanese female was referred to our hospital with gastrointestinal bleeding. Although small-bowel bleeding was suspected, no bleeding source was identified by enhanced computed tomography (CT), video capsule endoscopy (VCE), and double-balloon enteroscopy (DBE). Five years later, the patient had recurrent intermittent bloody stools with a significant decrease in hemoglobin levels. Although no active bleeding was observed on antegrade DBE, we detected a pulsatile submucosal uplift accompanied by a small red patch on the top of the uplift in the jejunum. Arteriovenous malformation (AVM) was suspected as the cause of small-bowel bleeding. Multiple-phase CT showed a number of small vascular ectasias during the arterial phase in the jejunum, and we confirmed the presence of multiple AVMs in the jejunum by selective angiography. To identify the location of the lesions and determine the minimal surgical margins, we performed intraoperative selective angiography with indocyanine green (ICG) injection. This technique allowed us to clearly observe the region and perform segmental small-bowel resection with minimal surgical margin. The patient reported that she has had no gastrointestinal bleeding at the two years follow-up visit.

## 1. Introduction

The source of bleeding in approximately 5% of all gastrointestinal bleeding cases is from the small bowel [1, 2]. Abnormal blood vessels of the small bowel cause 20% to 30% of cases of small-bowel bleeding [3–5]. Arteriovenous malformations (AVMs) are an important vascular cause of small-bowel bleeding. However, it is often difficult to diagnose and treat AVMs of the small-bowel. Furthermore, it is challenging to determine the optimal surgical margin of the lesions when there are multiple lesions.

Herein, we report a case of multiple AVMs of the small-bowel that were detected by double-balloon enteroscopy (DBE) and in which we determined the optimal surgical margins using intraoperative selective angiography with indocyanine green (ICG) injection.

## 2. Case Report

**Figure 1:**
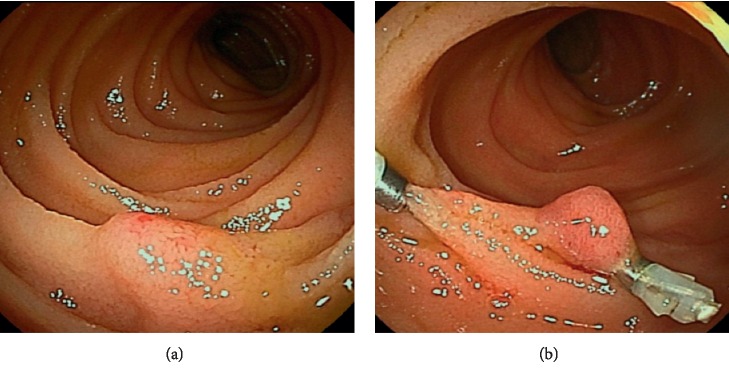
Antegrade double-balloon enteroscopy. A pulsatile submucosal uplift accompanied by a small red patch on the top of the uplift in the jejunum (a). Two clips were placed in close proximity to the submucosal uplift (b).

**Figure 2:**
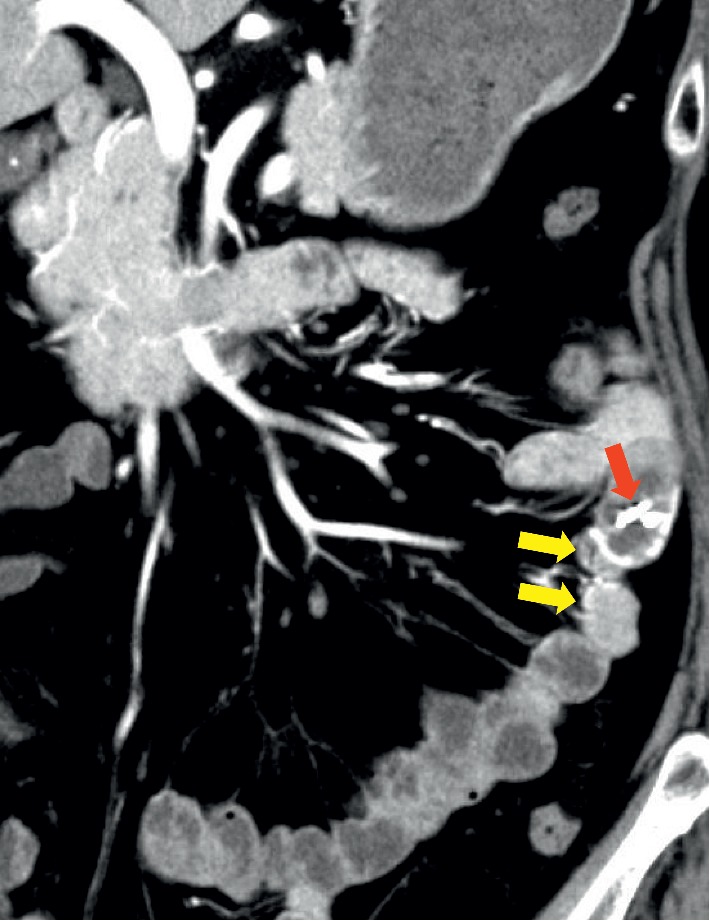
Multiple-phase computed tomography. Small vascular ectasias (yellow arrows) during the arterial phase were observed adjacent to the clips (red arrow).

**Figure 3:**
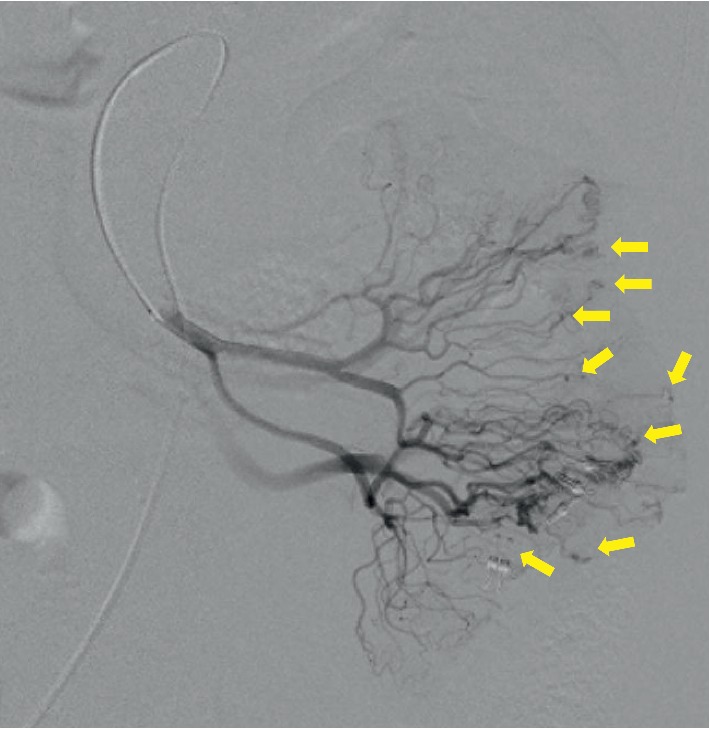
Selective angiography: multifocal niduses were detected in the jejunum and were supplied by the second jejunal arteries (yellow arrows).

**Figure 4:**
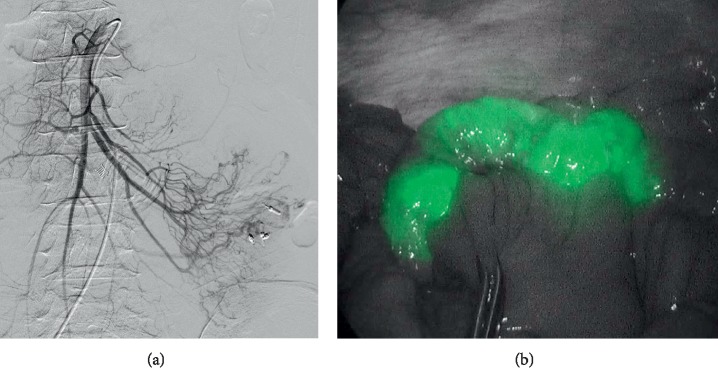
Intraoperative selective angiography with ICG injection: selective angiography from the second jejunal artery detected multifocal niduses in the jejunum (a). ICG was intraoperatively injected from the second jejunal artery. The region of ICG fluorescence was clearly recognized (b).

**Figure 5:**
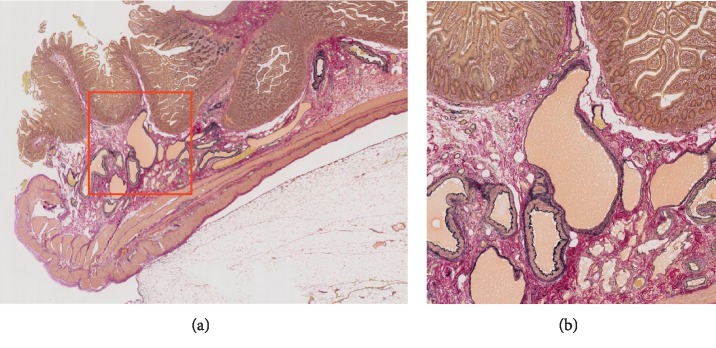
Histological findings: Elastica van Gieson staining revealed tortuous, dilated veins and arteries in the submucosal layer. The image in the right panel is a magnified view of the red box in the left panel. (a) ×magnifying glass imaging and (b) ×40.

A 62-year-old Japanese female was referred to our hospital with gastrointestinal bleeding five years prior to the current presentation. Five years before, esophagogastroduodenoscopy (EGD) and colonoscopy could not identify any abnormalities. As abdominal enhanced computed tomography (CT) with delayed phase imaging did not reveal a source of the bleeding, video capsule endoscopy (VCE) was carried out for small-bowel evaluation. Although blood clots were detected in the jejunum, no bleeding source was identified. Subsequently, antegrade DBE was performed for further investigation. However, no active bleeding was observed, and no bleeding sources were detected. Thereafter, she had recurrent bloody stools intermittently about once a year. Although small-bowel evaluations including VCE, enhanced CT, and DBE were performed repeatedly, the cause of small-bowel bleeding could not be identified. Her current presentation was marked by intermittent bloody stools and a fall in hemoglobin level to as low as 8.7 g/dl. VCE was immediately performed and showed blood clots localized to the proximal jejunum, which failed to show a source of bleeding. This was followed by antegrade DBE to evaluate the jejunum intensively. On DBE, a pulsatile submucosal uplift, which was accompanied by a small red patch on the top of the uplift without active bleeding, was revealed in the jejunum 30 cm on the anal side from the ligament of Treitz (Figure 1(a)). As an AVM was suspected as the cause of small-bowel bleeding, two clips were placed in close proximity to the submucosal uplift to control the arterial inflow for temporary bleeding prevention, which resulted in disappearance of pulsation and a color change by deterioration of blood flow in the lesion (Figure 1(b)). Multiple-phase CT revealed multiple small vascular ectasias during the arterial phase and their early draining veins adjacent to the clips that had been placed in the jejunum on DBE (Figure 2). As multiple AVMs were suspected, a selective angiography was employed to identify the location of the lesions. Multifocal niduses were detected adjacent to the clips in the jejunum supplied by the second jejunal arteries (Figure 3). To determine the minimal and optimal surgical margins, we performed intraoperative selective angiography with ICG injection. 2.5 mg/ml of ICG in a total volume of 0.2 ml was injected intraoperatively from the second jejunal artery via a selective angiographic microcatheter, which immediately stained a 30 cm segment of the jejunum (Figures 4(a) and 4(b)). ICG fluorescence enabled us to easily and clearly recognize the region and perform a segmental small-bowel resection with the minimal surgical margins. Pathological examination confirmed the presence of AVMs with features of tortuous, dilated veins, and arteries in the submucosal layer (Figure 5).

At the 2-year follow-up, the patient was doing well and denied any signs of small-bowel bleeding.

## 3. Discussion

Of all the sources of gastrointestinal bleeding, only a small percentage (5%) is attributed to a source in the small-bowel [1, 2]. In the small bowel, 20 to 30% of bleeding cases are caused by abnormal blood vessels, including angioectasias, AVMs, hemangiomas, and Dieulafoy's lesions [3–5]. Especially, AVMs are the most common cause of small-bowel bleeding in people over the age of 50 years.

It can be exceedingly difficult to localize and diagnose the small-bowel AVMs, particularly in cases of occult bleeding. For hemodynamically stable patients who are suspected of having small-bowel bleeding, after EGD and colonoscopy with normal results, VCE is recommended as the next diagnostic test, and device-assisted enteroscopy can be considered in patients with positive bleeding sources identified on VCE [6]. In general, AVMs in the small bowel have been described as submucosal uplift accompanied by redness, vascular proliferation, and vascular ectasia. However, most of them are findings in intraoperative endoscopy, and there are a limited number of cases diagnosed by preoperative endoscopy in the absence of active bleeding [7, 8]. The present case is a valuable report of AVMs in the small bowel that could be diagnosed by preoperative DBE, and the pulsatile submucosal uplift accompanied by a small red patch on the top might be an important finding that indicates AVMs. On the other hand, it is difficult to identify all the lesions by endoscopy in cases where multiple AVMs exist in the small bowel. In our case, we suspected the presence of multiple lesions based on the results of dynamic enhanced CT and confirmed it by selective angiography. In the diagnosis of AVM, dynamic enhanced CT and subsequent selective angiography as necessary to be performed, considering the possibility that there are multiple lesions.

With respect to the treatment of small-bowel AVMs, partial small-bowel resection with a minimal surgical margin is required for retaining small-bowel functions. However, it is exceedingly challenging to identify the location of lesions and determine the optimal surgical margin, especially in cases where there are multiple AVMs in the small bowel. A technique combining selective angiography with intraoperative methylene blue injection to aid the localization of obscure sources of GI bleeding, including AVMs, has been described in a number of case reports [9–11]. Although no reports described complications of this methylene blue injection technique, doses of 500 mg or greater of methylene blue can cause nausea, abdominal and chest pain, altered mental status, and methemoglobinemia [10, 12]. ICG is a nontoxic, near-infrared fluorophore, and its clinical applications have been recently proposed in various surgical settings. To the best of our knowledge, there has only been one reported case of intraoperative enteric mapping of small-bowel AVMs using ICG [13]. This new technique allows us to clearly visualize the regions of AVMs and immediately determine the optimal resection margins. Intraoperative selective angiography with ICG injection, which we performed in this case, is very safe and useful for the surgery of AVMs.

The optimal diagnosis and treatment strategy for multiple AVMs of the small bowel have not been fully elucidated. It has been reported that 5–37% of patients who underwent resection of AVMs will rebleed and one of the causes is incomplete excision [10, 14, 15]. The series of methods and techniques that we report here will contribute to the clinical treatment of AVMs of the small bowel.
